# Cluster Analysis of Finite Element Analysis and Bone Microarchitectural Parameters Identifies Phenotypes with High Fracture Risk

**DOI:** 10.1007/s00223-019-00564-7

**Published:** 2019-06-11

**Authors:** Leo D. Westbury, Clare Shere, Mark H. Edwards, Cyrus Cooper, Elaine M. Dennison, Kate A. Ward

**Affiliations:** 1 MRC Lifecourse Epidemiology Unit, University of Southampton, Southampton, UK; 2 Queen Alexandra Hospital, Portsmouth, UK; 3 NIHR Southampton Biomedical Research Centre, University of Southampton and University Hospital Southampton NHS Foundation Trust, Southampton, UK; 4 NIHR Oxford Biomedical Research Centre, University of Oxford, Oxford, UK; 5 Victoria University of Wellington, Wellington, New Zealand; 6 MRC Nutrition and Bone Health Research Group, Cambridge, UK

**Keywords:** Quantitative computed tomography, DXA, Epidemiology, Fracture, Osteoporosis, Finite element analysis

## Abstract

High-resolution peripheral quantitative computed tomography (HRpQCT) is increasingly used for exploring associations between bone microarchitectural and finite element analysis (FEA) parameters and fracture. We hypothesised that combining bone microarchitectural parameters, geometry, BMD and FEA estimates of bone strength from HRpQCT may improve discrimination of fragility fractures. The analysis sample comprised of 359 participants (aged 72–81 years) from the Hertfordshire Cohort Study. Fracture history was determined by self-report and vertebral fracture assessment. Participants underwent HRpQCT scans of the distal radius and DXA scans of the proximal femur and lateral spine. Poisson regression with robust variance estimation was used to derive relative risks for the relationship between individual bone microarchitectural and FEA parameters and previous fracture. Cluster analysis of these parameters was then performed to identify phenotypes associated with fracture prevalence. Receiver operating characteristic analysis suggested that bone microarchitectural parameters improved fracture discrimination compared to aBMD alone, whereas further inclusion of FEA parameters resulted in minimal improvements. Cluster analysis (k-means) identified four clusters. The first had lower Young modulus, cortical thickness, cortical volumetric density and Von Mises stresses compared to the wider sample; fracture rates were only significantly greater among women (relative risk [95%CI] compared to lowest risk cluster: 2.55 [1.28, 5.07], *p* = 0.008). The second cluster in women had greater trabecular separation, lower trabecular volumetric density and lower trabecular load with an increase in fracture rate compared to lowest risk cluster (1.93 [0.98, 3.78], *p* = 0.057). These findings may help inform intervention strategies for the prevention and management of osteoporosis.

**Electronic supplementary material:**

The online version of this article (10.1007/s00223-019-00564-7) contains supplementary material, which is available to authorized users.

## Introduction

Although measurements of areal bone mineral density (aBMD) of the hip or spine is considered the gold-standard for assessing fracture risk [1], it is widely recognised that there are limitations to only using DXA to determine bone fragility. Stratifying for fracture risk using DXA-derived aBMD may fail to identify some individuals at higher risk of fracture; indeed, as many as half of hip fractures occur in individuals with aBMD values considered low risk for osteoporotic fracture [2]. Prediction tools including clinical risk factors and DXA-derived BMD, such as the Fracture Risk Assessment Tool (FRAX^®^), improve prediction of fractures [3] but still fail to identify many who go on to sustain a fracture [4]. Understanding better the bone phenotypes associated with bone fragility may improve fracture prediction. To address this, techniques such as high-resolution peripheral quantitative computed tomography (HRpQCT) have been developed to explore volumetric bone mineral density (vBMD) and bone microarchitecture in both trabecular and cortical bones. It is now also possible to perform finite element analyses on HRpQCT scans and previous research has related tibial and radial FEA parameters to increased risk of fragility fractures at all sites, independent of aBMD [5–7]. Several other studies have reported HRpQCT parameters to be associated with prior or future fracture, independent of aBMD [7–11].

Our previous work in the Hertfordshire Cohort Study (HCS) used cluster analyses to identify two similar phenotypes associated with increased risk of prevalent fracture [12]. One demonstrated a ‘cortical deficiency’ phenotype, characterised by lower cortical thickness and cortical volumetric density and in men only, higher total and trabecular area. Men in this cluster had a normal aBMD and, therefore, may not have been identified as high risk by conventional DXA-based risk stratification alone. Men and women in the other cluster showed a ‘trabecular deficiency’ phenotype, with lower trabecular volumetric density and number. Similar radial clusters were identified in the Global Longitudinal Study of Osteoporosis in Women (GLOW) [13].

The aim of the current study was therefore to determine the extent to which bone microarchitectural and FEA parameters improve fracture discrimination compared to using aBMD alone. We hypothesised that combining bone microarchitectural parameters, geometry, BMD and FEA estimates of bone strength from HRpQCT as a composite of bone strength may improve discrimination of fragility fractures. The secondary aim was to repeat the cluster analyses, to see whether FEA parameters changed the identified phenotypes previously associated with fracture.

## Methods

### The Hertfordshire Cohort Study

The HCS comprises 2997 men and women born in Hertfordshire from 1931–1939 and who still lived there in 1998–2004 when they completed a baseline home interview and research clinic for a detailed characterisation of their health; the study has been described in detail previously [14]. At the baseline home interview (1998–2004), menopausal status (women only) and customary physical activity level (Dallosso questionnaire [15]) were ascertained by a nurse-administered questionnaire. Dietary calcium intake was determined using a food-frequency questionnaire [16]. Social class was coded from the 1990 OPCS Standard Occupational Classification (SOC90) unit group for occupation [17].

In 2011–2012, 570 East Hertfordshire participants were invited to take part in a further follow-up study; 376 agreed to participate [12]. Smoking status, alcohol consumption and whether participants had broken any bones since aged 45 years were ascertained by a nurse-administered questionnaire. Information was ascertained on whether participants had used bisphosphonates or hormone replacement therapy since HCS baseline.

The baseline HCS had ethical approval from the Hertfordshire and Bedfordshire Local Research Ethics Committee and the follow-up had ethical approval from the East and North Hertfordshire Ethical Committees. Investigations were conducted in accordance with the principles expressed in the Declaration of Helsinki.

### Participant Assessments at the 2011–2012 Follow-up

On the day of scanning, height was measured (wall-mounted SECA stadiometer) along with weight (calibrated SECA 770 digital floor scales, SECA Ltd, Hamburg) and used to derive BMI (kg/m^2^). Bilateral scans of the proximal femur were used for assessment of femoral neck aBMD (Lunar Prodigy Advance DXA scanner (GE Medical Systems)); the lowest value was used for analyses and diagnosis of osteoporosis or osteopenia. Morphometric vertebral fractures were diagnosed from a lateral spine view imaged using the same machine and graded based on the Genant semi-quantitative method of vertebral fracture assessment [18]. Participants with a vertebral fracture or a self-reported fracture since age 45 years were regarded as having had a previous fracture.

HRpQCT scans (XtremeCT, Scanco Medical AG, Switzerland) of the non-dominant distal radius were performed; dominant limbs were scanned if the non-dominant limb had previously fractured. In total, 110 parallel CT slices were obtained, representing a volume of bone 9 mm in axial length with a nominal resolution (voxel size) of 82 μm. The scan protocol was in accordance with manufacturer’s guidelines and as described by Boutroy et al. [19]. Using the method of Pauchard et al. [20], eight scans were excluded due to excessive motion artefact (grade 5 scans); scans of quality 4 and above were included in the analysis. Manufacturer standard evaluation and cortical porosity scripts were used for image analysis [5, 21–25]. Cortical and trabecular densities described in this study were ascertained using HRpQCT and are volumetric (mg/cm^3^).

A detailed description of the FEA analysis development for in vivo assessment of bone strength is in Boutroy et al. 2008 [25]. Briefly, the FE-solver (Image Processing Language) scripts were run to assess the biomechanical properties of the cortical and trabecular compartments and of the whole bone. The FEA models assume boundary conditions for an applied compressive load in the axial direction to the radius or tibia. From these the various stress, stiffness and failure load parameters were estimated.

### Statistical Analysis

Data were described using summary statistics. Age, anthropometric and lifestyle characteristics were compared between individuals who did and did not have a previous fracture. Skewed bone microarchitectural and FEA parameters were transformed prior to standardising. Poisson regression with robust variance estimation was used to derive relative risks for the relationship between individual parameters and previous fracture. Unadjusted and fully adjusted relative risks, accounting for age, height, BMI, dietary calcium, physical activity, smoking history (ever vs never), alcohol consumption, social class, bisphosphonate use, time since menopause (women only) and hormone replacement therapy (women only), were estimated.

Risk of previous fracture was examined using sex-specific logistic regression models containing the following sets of predictors: femoral neck aBMD only; femoral neck aBMD and bone microarchitectural parameters that were significantly associated with fracture in fully adjusted analysis; FEA parameters that were significantly associated with fracture in sex-specific analysis as additional predictors. The following pairs of predictors were highly collinear and, therefore, were not included in the same model: trabecular and total area; bone stiffness and failure load; trabecular strain and von Mises stresses (trabecular). Performance of models was assessed using the areas under the receiver operating characteristic curves (AUC).

A cluster analysis of the complete set of bone microarchitectural and FEA parameters was performed using the k-means partitioning method. The number of clusters selected was based on the stability of the clustering, and on the potential for identifying contrasting phenotypes as in previous analyses [12, 13]. The means and standard deviations (SD) of the standardised parameters, and fracture proportion were then determined for each cluster. Poisson regression with robust variance estimation was used to determine the likelihood of fracture in each cluster compared to the lowest risk cluster. Mean femoral neck aBMD in each cluster was compared to the cluster with the lowest fracture risk.

The analysis sample comprised of 359 participants with complete data for all radial bone microarchitectural and FEA parameters. Healthy participant effects were assessed by comparing HCS baseline participant characteristics between this analysis sample of 359 participants and the group of 2638 participants who attended the HCS baseline clinic but were not included in the analysis sample. All analyses were performed among men and women separately using Stata 15 (StataCorp. 2017. Stata Statistical Software: Release 15. College Station, TX: StataCorp LLC).

## Results

### Participant Characteristics

The characteristics of the study population are presented in Table 1. Mean (SD) age of the 359 participants at the time of scan was 76.3 (2.6) years. Overall 45 (26.2%) men and 50 (31.6%) women had a previous fracture (vertebral or self-reported since age 45 years). Locations of fractures in this cohort have been described previously [12].

**Table 1:** Participant characteristics of the analysis sample

	Men (*n* = 188)	Women (*n* = 171)	Obs
	Mean (SD)		
Age (years)	76.1 (2.5)	76.5 (2.7)	359
Time since menopause (years)	NA	28.1 (6.6)	168/171
Height (cm)	173.4 (6.7)	159.9 (5.8)	359
Weight (kg)	82.5 (12.2)	71.2 (12.7)	359
BMI (kg/m^2^)	27.4 (3.8)	27.8 (4.7)	359
Weekly dietary calcium (g)	8.6 (2.1)	7.9 (2.6)	359
Physical activity score (Dallosso)	65.6 (13.5)	62.1 (13.8)	359
Femoral neck aBMD (g/cm^2^)	0.94 (0.14)	0.83 (0.12)	345

	Men (*n* = 188)	Women (*n* = 171)	Obs
	*N* (%)		
Smoking (ever)	103 (58.5%)	60 (37.7%)	335
High alcohol consumption	27 (15.3%)	3 (1.9%)	335
Social class (manual)	100 (55.6%)	96 (56.1%)	351
Bisphosphonates (since baseline)	9 (5.1%)	32 (19.9%)	337
Hormone replacement therapy (since baseline)	NA	37 (23.0%)	161/171
Osteoporosis (FN t-score < -2.5)	13 (7.2%)	17 (10.3%)	345
Osteopenia (-2.5 ≤ FN t-score < -1)	83 (46.1%)	86 (52.1%)	345
Self-reported fracture since 45 years	40 (23.3%)	44 (27.7%)	331
Vertebral fracture	10 (5.4%)	14 (8.3%)	354
Any fracture (self-reported or vertebral)*	45 (26.2%)	50 (31.6%)	330

High alcohol consumption was defined as > 21 units per week for men and > 14 units per week for women

Dietary calcium, social class and physical activity were ascertained at HCS baseline (1998–2004). All other characteristics were ascertained in 2011–2012

*Obs* number of non-missing observations; *FN* femoral neck; *aBMD* areal bone mineral density

*Locations of fractures in this cohort have been described previously [12]

On average, women who had a previous fracture were older than those who did not (77.3 years vs. 76.1 years, *p* = 0.005); no significant differences were observed among men. Among men and women, the following characteristics did not differ significantly between those who did and did not have a fracture: height, weight, dietary calcium, physical activity, smoking status and alcohol consumption (data not shown).

### Assessing Healthy Participant Effects in the Analysis Sample

Compared to the 2638 participants who attended the HCS baseline clinic but were not included in the analysis sample, both men and women in the analysis sample had higher self-reported physical activity at baseline (*p* < 0.006); only men in the analysis sample were more likely to have never smoked at baseline (*p* = 0.003) and only women were less likely to have high alcohol consumption (*p* = 0.047). The proportion who were of manual social class (classes IIIM, IV and V) did not differ significantly (*p* > 0.05) between the two groups.

### Relationships Between Bone Microarchitectural and FEA Parameters and Previous Fracture

The associations between individual bone microarchitectural and FEA parameters and previous fracture among men and women are presented in Table 2. Among men, higher total and trabecular area and lower cortical thickness were each associated with increased risk of previous fracture in unadjusted and adjusted analyses (*p* < 0.03); no FEA parameters were associated with fracture risk (*p* > 0.05). Among women, lower values of the following parameters were associated with increased risk of fracture in unadjusted and adjusted analyses (*p* < 0.04): cortical area and porosity; trabecular density, thickness, Von Mises stresses and strain; bone stiffness and failure load and Young Modulus.

**Table 2:** Relative risks for previous fracture per standard deviation increase in parameter

	Men				Women			
Parameter	Unadjusted		Adjusted*		Unadjusted		Adjusted*	
	RR (95% CI)	*P* value	RR (95% CI)	*P* value	RR (95% CI)	*P* value	RR (95% CI)	*P* value
Bone microarchitectural								
Total area	**1.48 (1.13**, **1.93)**	**0.004**	**1.48 (1.11**, **1.99)**	**0.009**	1.00 (0.79, 1.26)	0.983	0.86 (0.64, 1.16)	0.321
Cortical area	0.85 (0.66, 1.08)	0.189	0.84 (0.65, 1.09)	0.201	**0.69 (0.54**, **0.86)**	**0.001**	**0.69 (0.52**, **0.92)**	**0.012**
Cortical thickness	**0.72 (0.56**, **0.92)**	**0.009**	**0.74 (0.57**, **0.95)**	**0.021**	**0.72 (0.57**, **0.91)**	**0.007**	0.76 (0.58, 1.01)	0.057
Cortical density	0.84 (0.66, 1.08)	0.173	0.81 (0.62, 1.05)	0.115	0.83 (0.66, 1.05)	0.118	0.91 (0.70, 1.18)	0.472
Cortical porosity	0.90 (0.70, 1.16)	0.418	0.91 (0.68, 1.21)	0.507	**0.77 (0.61**, **0.98)**	**0.030**	**0.77 (0.62**, **0.96)**	**0.018**
Trabecular area	**1.47 (1.17**, **1.85)**	**0.001**	**1.46 (1.12**, **1.91)**	**0.005**	1.06 (0.85, 1.33)	0.591	0.94 (0.70, 1.26)	0.670
Trabecular density	0.82 (0.63, 1.06)	0.130	0.78 (0.61, 1.00)	0.053	**0.66 (0.54**, **0.79)**	**<0.001**	**0.78 (0.61**, **0.98)**	**0.033**
Trabecular number	0.82 (0.63, 1.06)	0.126	0.79 (0.62, 1.01)	0.055	**0.75 (0.60**, **0.92)**	**0.007**	0.89 (0.70, 1.13)	0.346
Trabecular thickness	0.86 (0.65, 1.13)	0.269	0.82 (0.62, 1.08)	0.153	**0.66 (0.52**, **0.82)**	**<0.001**	**0.70 (0.55**, **0.90)**	**0.004**
Trabecular separation	1.23 (0.96, 1.60)	0.107	**1.28 (1.01**, **1.62)**	**0.039**	**1.38 (1.13**, **1.70)**	**0.002**	1.16 (0.91, 1.46)	0.230
FEA								
Bone stiffness	0.98 (0.74, 1.30)	0.889	0.94 (0.70, 1.27)	0.690	**0.60 (0.48**, **0.76)**	**<0.001**	**0.65 (0.51**, **0.83)**	**0.001**
Bone failure load	1.01 (0.76, 1.34)	0.951	0.97 (0.71, 1.31)	0.831	**0.59 (0.47**, **0.75)**	**<0.001**	**0.64 (0.50**, **0.82)**	**<0.001**
% load trabecular (distal)	1.27 (0.97, 1.67)	0.082	1.23 (0.93, 1.62)	0.143	1.09 (0.85, 1.40)	0.488	1.07 (0.79, 1.44)	0.662
% load trabecular (proximal)	1.13 (0.89, 1.44)	0.307	1.05 (0.83, 1.33)	0.675	0.96 (0.74, 1.23)	0.721	0.99 (0.77, 1.28)	0.966
Young modulus	0.78 (0.60, 1.01)	0.057	0.76 (0.56, 1.02)	0.065	**0.66 (0.54**, **0.82)**	**<0.001**	**0.73 (0.56**, **0.95)**	**0.019**
Von Mises stresses (trabecular)	0.94 (0.72, 1.24)	0.672	0.89 (0.66, 1.19)	0.424	**0.71 (0.55**, **0.92)**	**0.009**	**0.74 (0.56**, **0.96)**	**0.024**
Von Mises stresses (cortical)	0.95 (0.73, 1.24)	0.724	0.95 (0.72, 1.25)	0.713	1.00 (0.78, 1.27)	0.970	1.01 (0.79, 1.28)	0.963
Trabecular strain	0.92 (0.70, 1.22)	0.576	0.87 (0.65, 1.17)	0.358	**0.71 (0.55**, **0.92)**	**0.009**	**0.74 (0.57**, **0.96)**	**0.022**
Cortical strain	0.97 (0.77, 1.22)	0.795	0.95 (0.73, 1.24)	0.719	0.90 (0.71, 1.15)	0.397	0.93 (0.74, 1.16)	0.528

Relative risks (RR) were obtained from Poisson regression models with robust variance estimation

Significant relative risks (*P* < 0.05) are given in bold

*FEA* finite element analysis; *BMD* bone mineral density

*Adjusted for age, height, BMI, dietary calcium, physical activity, smoking history (ever vs. never), alcohol consumption, social class, bisphosphonate use, time since menopause (women only) and hormone replacement therapy (women only)

### Comparison of Fracture Prediction Models Using Receiver Operating Characteristic Analysis

Areas under the receiver operating characteristic curves (AUC) for different models used to examine risk of fracture are presented in Table 3. Including bone microarchitectural parameters increased the AUC values compared to models based only on femoral neck aBMD (0.67 [95% CI: 0.60, 0.75] vs. 0.61 [0.52, 0.68] for men; 0.76 [0.69, 0.83] vs. 0.70 [0.63, 0.78] for women). However, these increases in AUC values were not statistically significant (*p* > 0.05). Among women, additionally including FEA parameters only resulted in small increases in AUC values. The effect on fracture discrimination of additionally including FEA parameters among men was not examined as no FEA parameters were associated with the risk of previous fracture among men. Sex-specific Pearson correlations between the parameters in Table 3 are stated in Online Appendix 1.

**Table 3:** Receiver operating characteristic analysis of models predicting previous fracture based on combinations of BMD, bone microarchitectural and FEA parameters

Model	Predictors	AUC (95% CI)
Men		
1	Femoral neck aBMD	0.61 (0.52, 0.68)
2	Femoral neck aBMD	0.67 (0.60, 0.75)
	Total area	
	Cortical thickness	
	Trabecular separation	
Women		
1	Femoral neck aBMD	0.70 (0.63, 0.78)
2	Femoral neck aBMD	0.76 (0.69, 0.83)
	Cortical area	
	Cortical porosity	
	Trabecular density	
	Trabecular thickness	
3	Femoral neck aBMD	0.78 (0.70, 0.84)
	Cortical area	
	Cortical porosity	
	Trabecular density	
	Trabecular thickness	
	Bone failure load	
	Young modulus	
	Von Mises stresses (trabecular)	

*aBMD* areal bone mineral density; *FEA* finite element analysis; *AUC* area under receiver operator characteristic curve

### Cluster Analysis of Bone Microarchitectural and FEA Radius Parameters

**Fig. 1:**
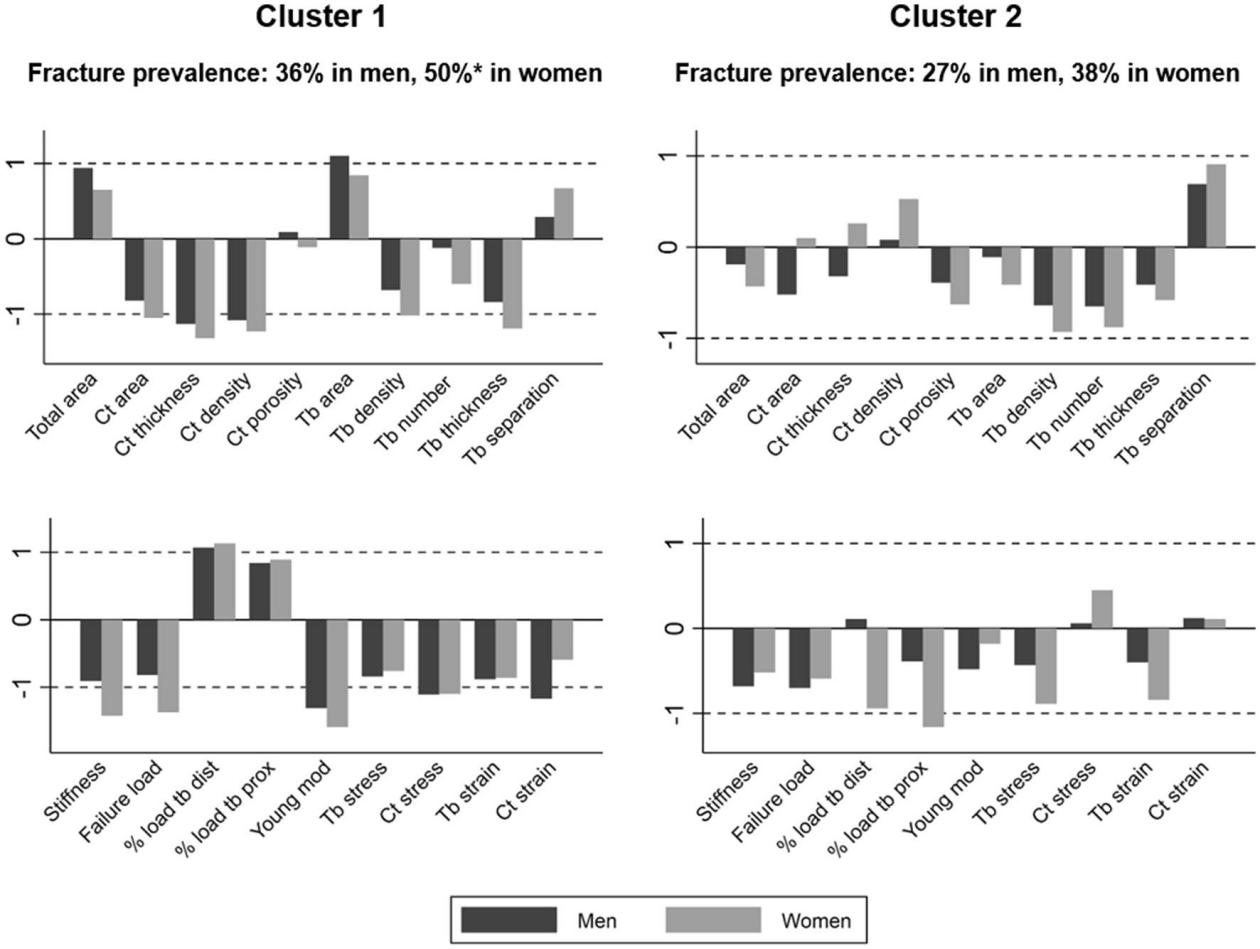
Means of standardised radial parameters within Clusters 1 and 2. *Significantly higher fracture prevalence (*p* = 0.008) compared to Cluster 4 (lowest risk cluster). *Ct* cortical; *Tb* trabecular; *Ct density* cortical density; *Tb density* trabecular density; *dist* distal; *prox* proximal; *mod* modulus; *Tb stress* Von Mises stresses (trabecular); *Ct stress* Von Mises stresses (cortical)

Four clusters were obtained among men and women. Summary statistics of the standardised bone microarchitectural and FEA parameters, femoral neck aBMD and fracture prevalence according to the different clusters are illustrated in Tables 4 and 5. Figure 1 displays means of the standardised parameters and fracture prevalence for Clusters 1 and 2, the highest risk clusters. Unless otherwise indicated, statements about the bone microarchitectural and FEA parameters refer to sex-specific means which differed by more than one SD compared to the analysis sample; comparisons of aBMD and fracture prevalence are in relation to Cluster 4, the cluster with lowest fracture risk. Men in Cluster 4 had higher cortical thickness and Young modulus and lower trabecular area; women had greater Young modulus and stiffness (all differences in means > 0.9 SD).

**Table 4:** Mean (SD) parameters within each cluster analysis group among men

	Cluster 1 (*n* = 36)	Cluster 2 (*n* = 51)	Cluster 3 (*n* = 49)	Cluster 4 (*n* = 52)
Bone microarchitectural (standardised)				
Total area	0.94 (0.73)	−0.19 (0.84)	0.37 (0.69)	−0.82 (0.83)
Cortical area	−0.82 (0.89)	−0.52 (0.74)	0.26 (0.77)	0.82 (0.73)
Cortical thickness	**−1.13 (0.70)**	−0.32 (0.55)	0.03 (0.64)	**1.07 (0.70)**
Cortical density	**−1.08 (0.76)**	0.08 (0.74)	-0.15 (0.79)	0.81 (0.79)
Cortical porosity	0.09 (0.80)	−0.39 (0.94)	0.52 (0.99)	−0.17 (1.00)
Trabecular area	**1.10 (0.78)**	−0.11 (0.73)	0.28 (0.72)	−0.92 (0.67)
Trabecular density	−0.68 (0.86)	−0.64 (0.68)	0.85 (0.65)	0.30 (0.88)
Trabecular number	−0.12 (0.94)	−0.65 (0.84)	0.57 (0.88)	0.18 (0.93)
Trabecular thickness	−0.84 (0.88)	−0.41 (0.77)	0.75 (0.64)	0.28 (0.94)
Trabecular separation	0.29 (0.88)	0.69 (0.79)	−0.70 (0.85)	−0.22 (0.89)
FEA (standardised)				
Bone stiffness	−0.91 (0.84)	−0.68 (0.54)	0.90 (0.72)	0.44 (0.61)
Bone failure load	−0.82 (0.87)	−0.70 (0.57)	0.92 (0.73)	0.39 (0.62)
% load trabecular (distal)	**1.07 (0.55)**	0.11 (0.67)	0.32 (0.52)	**−1.15 (0.70)**
% load trabecular (proximal)	0.84 (0.94)	−0.39 (0.76)	0.59 (0.57)	−0.76 (0.78)
Young modulus	**−1.31 (0.62)**	−0.48 (0.39)	0.49 (0.57)	0.92 (0.65)
Von Mises stresses (trabecular)	−0.84 (0.71)	−0.43 (0.80)	0.80 (0.65)	0.25 (0.97)
Von Mises stresses (cortical)	**−1.11 (1.01)**	0.06 (0.77)	0.03 (0.76)	0.69 (0.68)
Trabecular strain	−0.88 (0.74)	−0.40 (0.84)	0.77 (0.61)	0.27 (0.94)
Cortical strain	**−1.17 (1.14)**	0.12 (0.68)	0.07 (0.81)	0.62 (0.58)
DXA				
Femoral neck aBMD (g/cm^2^)	0.88 (0.12)	0.87 (0.11)	1.02 (0.14)	0.98 (0.12)
*Pvalue*	*<* *0.001*	*<* *0.001*	*0.108*	Reference
Normal*	13 (37.1%)	12 (25.0%)	33 (71.7%)	26 (51.0%)
Osteopenic*	16 (45.7%)	30 (62.5%)	13 (28.3%)	24 (47.1%)
Osteoporosis*	6 (17.1%)	6 (12.5%)	0 (0.0%)	1 (2.0%)
Fracture				
Prevalent fracture*	12 (36.4%)	13 (27.1%)	12 (26.1%)	8 (17.8%)
Relative risk of fracture	2.05 (0.94,4.44)	1.52 (0.70,3.33)	1.47 (0.66,3.26)	Reference
*Pvalue*	*0.071*	*0.292*	*0.346*	Reference
Vertebral fracture*	3 (8.3%)	4 (8.0%)	1 (2.1%)	2 (3.8%)

Cluster analysis was performed on the combined set of bone microarchitectural and FEA parameters

Bold if mean > 1 standard deviation from sample mean

*P* values for relative risk of prevalent fracture were calculated using Poisson regression with robust variance estimation. *P* values for differences in femoral neck aBMD were calculated using linear regression. *P* values are for differences compared to Cluster 4 (lowest risk)

*N (%)

**Table 5:** Mean (SD) parameters within each cluster analysis group among women

	Cluster 1 (*n* = 23)	Cluster 2 (*n* = 39)	Cluster 3 (*n* = 47)	Cluster 4 (*n* = 62)
Bone microarchitectural (standardised)				
Total area	0.65 (0.89)	**−**0.43 (0.95)	0.60 (0.88)	**−**0.42 (0.78)
Cortical area	**−1.05 (0.76)**	0.10 (0.88)	**−**0.44 (0.80)	0.66 (0.77)
Cortical thickness	**−1.32 (0.59)**	0.26 (0.72)	**−**0.61 (0.58)	0.79 (0.68)
Cortical density	**−1.23 (0.87)**	0.53 (0.73)	**−**0.54 (0.66)	0.53 (0.77)
Cortical porosity	−0.11 (1.08)	**−**0.63 (0.91)	0.24 (0.87)	0.25 (0.96)
Trabecular area	0.84 (0.95)	**−**0.41 (0.80)	0.67 (0.86)	**−**0.55 (0.68)
Trabecular density	**−1.02 (0.59)**	**−**0.93 (0.70)	0.09 (0.50)	0.89 (0.65)
Trabecular number	**−**0.60 (0.80)	**−**0.88 (0.75)	0.19 (0.82)	0.63 (0.81)
Trabecular thickness	**−1.19 (0.72)**	**−**0.58 (0.88)	0.04 (0.68)	0.78 (0.64)
Trabecular separation	0.67 (0.72)	0.91 (0.75)	**−**0.15 (0.73)	**−**0.71 (0.79)
FEA (standardised)				
Bone stiffness	**−1.42 (0.37)**	**−**0.52 (0.57)	**−**0.09 (0.63)	0.92 (0.66)
Bone failure load	**−1.37 (0.40)**	**−**0.59 (0.57)	**−**0.01 (0.65)	0.89 (0.68)
% load trabecular (distal)	**1.13 (0.42)**	**−**0.94 (0.74)	0.78 (0.52)	**−**0.42 (0.69)
% load trabecular (proximal)	0.89 (0.88)	**−1.16 (0.73)**	0.66 (0.75)	**−**0.10 (0.48)
Young modulus	**−1.59 (0.37)**	**−**0.18 (0.64)	**−**0.40 (0.42)	1.00 (0.48**)**
Von Mises stresses (trabecular)	**−**0.76 (0.55)	**−**0.89 (0.79)	0.17 (0.80)	0.72 (0.73)
Von Mises stresses (cortical)	**−1.10 (1.20)**	0.45 (0.68)	**−**0.34 (0.90)	0.38 (0.74)
Trabecular strain	**−**0.86 (0.49)	**−**0.84 (0.85)	0.14 (0.75)	0.74 (0.72)
Cortical strain	**−**0.59 (1.71)	0.11 (0.64)	0.08 (1.15)	0.09 (0.57)
DXA				
Femoral neck aBMD (g/cm^2^)	0.75 (0.09)	0.79 (0.11)	0.80 (0.10)	0.90 (0.12)
*Pvalue*	*<* *0.001*	*<* *0.001*	*<* *0.001*	Reference
Normal*	3 (13.0%)	10 (27.0%)	10 (21.7%)	39 (66.1%)
Osteopenic*	16 (69.6%)	20 (54.1%)	32 (69.6%)	18 (30.5%)
Osteoporosis*	4 (17.4%)	7 (18.9%)	4 (8.7%)	2 (3.4%)
Fracture				
Prevalent fracture*	10 (50.0%)	14 (37.8%)	15 (33.3%)	11 (19.6%)
Relative risk of fracture	2.55 (1.28,5.07)	1.93 (0.98,3.78)	1.70 (0.86,3.33)	Reference
*Pvalue*	*0.008*	*0.057*	*0.124*	Reference
Vertebral fracture*	3 (13.6%)	4 (10.8%)	3 (6.4%)	4 (6.5%)

Cluster analysis was performed on the combined set of bone microarchitectural and FEA parameters

Bold if mean > 1 standard deviation from sample mean

*P* values for relative risk of prevalent fracture were calculated using Poisson regression with robust variance estimation. *P* values for differences in femoral neck aBMD were calculated using linear regression. *P* values are for differences compared to Cluster 4 (lowest risk)

*N (%)

In Cluster 1, lower means of the following parameters were found among men and women: cortical thickness, density, Young modulus and Von Mises stresses. Among men only, cortical strain was also lower and among women only, Cluster 1 was associated with lower cortical area, trabecular density and thickness, bone stiffness and failure load. Fracture risk was only significantly greater among women (relative risk [95% CI]: 2.55 [1.28, 5.07], *p* = 0.008).

In Cluster 2, a trabecular deficient phenotype among women only was found, with higher trabecular separation, lower trabecular density and lower proportion of load applied to both distal and proximal trabecular bones (differences in means > 0.9 SD). This phenotype was associated with increased fracture risk in women (1.93 [0.98, 3.78], *p* = 0.057).

In Cluster 3, bone stiffness and failure load were higher among men only (differences in means ≥ 0.9 SD), though aBMD was not different to Cluster 4 (*p* = 0.108). No significant differences regarding fracture risk were observed.

## Discussion

This study assessed the additional utility of adding HRpQCT, BMD, microarchitectural and FEA parameters to enhance fracture discrimination in a cohort of elderly men and women. Whilst individual measures of bone microarchitecture and FEA discriminated fracture cases versus non-fracture groups, and were selected in cluster analyses, little benefit of adding the FEA parameters was found over and above femoral neck aBMD and bone microarchitecture in terms of fracture discrimination.

In terms of individual bone microarchitectural parameters, higher total and trabecular area and lower cortical thickness were associated with increased fracture risk in unadjusted and adjusted analyses among men; corresponding parameters among women were lower cortical area and porosity, trabecular density and thickness. The cortical porosity result in women seems a little counterintuitive though is consistent with previous work in this cohort [12], and in GLOW [13]. The observation may be due to the way the cortical porosity analysis script defines a pore [26] which is based on how many neighbouring voxels have a similar low attenuation value. If an individual has less cortical bone and thinner cortices (as shown in our current analyses), it may be that fewer pores meet these criteria, which would be reflected in a lower  % porosity. Secondly, the bone of participants with fracture may have a lower turnover, and thus repair rate, which may also result in fewer pores and an increase in fracture risk. In men, no FEA parameters were associated with fracture risk, but in women, lower bone stiffness, failure load, Young modulus and trabecular stress and strain were all associated with higher fracture risk in both unadjusted and adjusted analyses.

It appears that men at higher risk of fracture had larger bones with a thinner cortex and deterioration of trabecular bone, which was not translated into reduced bone strength as measured by linear FEA. Linear FEA has been criticised as it only evaluates stresses and strains placed on the bone in one direction, representing a linear compressive force on the bone, whereas non-linear FEA may provide additional information on fracture risk [27]. Perhaps this phenotype in men may produce vulnerability to forces placed on bones from other directions, including bending, which would not be identified by linear FEA. If this were the case, it may be expected that these men may be at higher risk of hip or radial fractures, frequently sustained on falling, compared with vertebral, classically compressive, fractures. During ageing, men also compensate for bone loss with greater periosteal formation to a greater extent than women [28], which may protect against compressive forces better in males. Conversely, the impaired linear bone strength parameters seen in women may suggest these women are more prone to compressive fractures. Vertebral fractures were the most common fracture among both males and females, although due to the small number of fractures in our cohort, comparison of fracture types in different clusters was not possible. It would be interesting to investigate this further, using non-linear FEA, in a larger sample with larger numbers of fractures.

The ROC curve analyses showed the benefit of adding relevant bone microarchitectural parameters into DXA-derived femoral neck aBMD assessment of fracture risk. This benefit was not statistically significant, but this may be influenced by our small sample size. Despite the association of FEA parameters with fracture risk in females, ROC curve analysis revealed FEA had little additional benefit in predicting fracture over models using bone microarchitectural parameters and aBMD. FEA is computationally intensive, and therefore time consuming and expensive. Our analyses suggest the additional information may not justify its use in predicting fractures in clinical practice. These findings are in contrast with previous studies which have found FEA to be a valuable predictor of prevalent and incident fracture, more important than other HRpQCT measures [5–7, 29]. One study found a machine learning model incorporating HRpQCT measures could predict fractures better than aBMD [30]. Another study reported the additional benefit of a combination of a trabecular and cortical parameter or FEA failure load to femoral neck aBMD or FRAX-BMD in predicting fracture in postmenopausal women. However, failure load could be replaced with aBMD of the ultra-distal radius with no significant reduction in predictive worth [24]. This is similar to our findings, with the addition of FEA providing little additional benefit over bone microarchitectural parameters.

Our cluster analysis revealed similar clusters to previous findings [12] with Cluster 1 showing a predominantly ‘cortical deficiency’ phenotype based on bone microarchitectural parameters. In males, trabecular area was greater, perhaps reflecting a greater proportion of trabecular bone due to a reduction in cortical bone. In females, trabecular density and thickness were also lower, suggestive of more generalised deterioration of bone structure. FEA in this cluster showed greater percentage of load on trabecular bone, lower Young modulus and lower cortical stresses in both males and females and lower bone stiffness and bone failure load in females. Compared to the lowest risk cluster, this cluster also had lower mean aBMD, and was the only cluster with significantly greater fracture risk in females.

The trabecular phenotype was less definitive than in our previous analysis but was indicated in Cluster 2 where females tended towards a ‘trabecular deficiency’ phenotype, and tended towards higher fracture risk, although this did not reach statistical significance. This is likely due to the additional FEA parameters included in the cluster analysis compared to the previous analysis which only included bone microarchitectural parameters. Bone microarchitectural and FEA parameters were similar to the wider sample, even though there was a significantly lower aBMD in both males and females compared to the lowest risk cluster.

These findings demonstrate the importance of HRpQCT parameters and identify cortical deterioration as a key contributor to fracture risk. As with our findings, previous studies have found both cortical and trabecular microarchitectural deterioration important for fracture risk, some finding cortical [31, 32], and some finding trabecular [9, 19, 33] changes more important. One study found both lower trabecular and cortical volumetric density were independently associated with fracture incidence [24]. Our use of cluster analysis helps to elucidate different phenotypes within the population at risk of fractures, with different contributions of both trabecular and cortical parameters.

The strengths of our study include basing our analyses on the HCS, a well characterised cohort where data were rigorously collected by an experienced multidisciplinary team. Furthermore, our analyses used both bone microarchitectural and FEA parameters to provide a comprehensive illustration of bone phenotypes.

This study has some limitations. Firstly, a healthy responder bias has been observed in HCS and examining participant characteristics according to inclusion status has revealed healthier lifestyles at baseline for participants included in the analysis sample compared to those who were not. However, our analyses were internal, so bias would only arise if the associations of interest differed systematically between those who were included in the analysis sample and those who were not; this seems unlikely. Secondly, temporal causation cannot be inferred as our study has a cross-sectional design. It may be that the differences in bone microstructure seen are secondary to remodelling in response to fracture, rather than properties of the bone which predispose to fracture, especially as we have only collected information about previous fractures. Thirdly, fracture status was missing for some participants, although this information was available for the vast majority (91.9%) of the analysis sample. Finally, the low numbers of reported fractures and a relatively small sample size, along with the lack of stability regarding cluster analysis algorithms in general, may limit the generalisability of findings. However, the similarity of the clusters observed to those in other analyses and their biological plausibility suggests that they are robust.

In conclusion, microarchitectural deterioration, bone geometry and, in women, FEA-derived bone strength contributed to an increased risk of previous fracture. Cluster analysis revealed a cortical and a trabecular deficiency phenotype, which both showed lower aBMD in men and women. Only women with the cortical deficiency phenotype had significantly increased risk of previous fractures. In this cohort, adding bone microarchitectural parameters to aBMD could better predict previous fracture, but further addition of FEA conferred little benefit.

## Electronic supplementary material

Below is the link to the electronic supplementary material.

Supplementary material 1 (DOCX 15 kb)

## References

[1] Marshall D, Johnell O, Wedel H (1996). Meta-analysis of how well measures of bone mineral density predict occurrence of osteoporotic fractures. BMJ.

[2] Wainwright SA, Marshall LM, Ensrud KE, Cauley JA, Black DM, Hillier TA, Hochberg MC, Vogt MT, Orwoll ES (2005). Hip fracture in women without osteoporosis. J Clin Endocrinol Metab.

[3] Kanis JA, Oden A, Johnell O, Johansson H, De Laet C, Brown J, Burckhardt P, Cooper C, Christiansen C, Cummings S, Eisman JA, Fujiwara S, Gluer C, Goltzman D, Hans D, Krieg MA, La Croix A, McCloskey E, Mellstrom D, Melton LJ, Pols H, Reeve J, Sanders K, Schott AM, Silman A, Torgerson D, van Staa T, Watts NB, Yoshimura N (2007). The use of clinical risk factors enhances the performance of BMD in the prediction of hip and osteoporotic fractures in men and women. Osteoporos Int.

[4] Jiang X, Gruner M, Tremollieres F, Pluskiewicz W, Sornay-Rendu E, Adamczyk P, Schnatz PF (2017). Diagnostic accuracy of FRAX in predicting the 10-year risk of osteoporotic fractures using the USA treatment thresholds: a systematic review and meta-analysis. Bone.

[5] Vilayphiou N, Boutroy S, Szulc P, van Rietbergen B, Munoz F, Delmas PD, Chapurlat R (2011). Finite element analysis performed on radius and tibia HR-pQCT images and fragility fractures at all sites in men. J Bone Miner Res.

[6] Vilayphiou N, Boutroy S, Sornay-rendu E, Munoz F, Delmas PD, Chapurlat R (2010). Finite element analysis performed on radius and tibia HR-pQCT images and fragility fractures at all sites in postmenopausal women. Bone.

[7] Fink HA, Langsetmo L, Vo TN, Orwoll ES, Schousboe JT, Ensrud KE (2018). Association of high-resolution peripheral quantitative computed tomography (HR-pQCT) bone microarchitectural parameters with previous clinical fracture in older men: the osteoporotic fractures in men (MrOS) study. Bone.

[8] Sornay-Rendu E, Boutroy S, Munoz F, Delmas PD (2007). Alterations of cortical and trabecular architecture are associated with fractures in postmenopausal women, partially independent of decreased BMD measured by DXA: the OFELY study. J Bone Miner Res.

[9] Sornay-Rendu E, Boutroy S, Duboeuf F, Chapurlat RD (2017). Bone microarchitecture assessed by HR-pQCT as predictor of fracture risk in postmenopausal women: the OFELY study. J Bone Miner Res.

[10] Szulc P, Boutroy S, Vilayphiou N, Chaitou A, Delmas PD, Chapurlat R (2011). Cross-sectional analysis of the association between fragility fractures and bone microarchitecture in older men: the STRAMBO study. J Bone Miner Res.

[11] Boutroy S, Khosla S, Sornay-Rendu E, Zanchetta MB, McMahon DJ, Zhang CA, Chapurlat RD, Zanchetta J, Stein EM, Bogado C, Majumdar S, Burghardt AJ, Shane E (2016). Microarchitecture and peripheral BMD are impaired in postmenopausal white women with fracture independently of total hip T-Score: an International Multicenter Study. J Bone Miner Res.

[12] Edwards M, Robinson D, Ward K, Javaid M, Walker-Bone K, Cooper C, Dennison E (2016). Cluster analysis of bone microarchitecture from high resolution peripheral quantitative computed tomography demonstrates two separate phenotypes associated with high fracture risk in men and women. Bone.

[13] Litwic A, Westbury L, Robinson D, Ward K, Cooper C, Dennison E (2018). Bone phenotype assessed by HRpQCT and associations with fracture risk in the GLOW study. Calcif Tissue Int.

[14] Syddall H, Sayer A, Dennison E, Martin H, Barker D, Cooper C (2005). Cohort profile: the hertfordshire cohort study. Int J Epidemiol.

[15] Dallosso H, Morgan K, Bassey E, Ebrahim S, Fentem P, Arie T (1988). Levels of customary physical activity among the old and the very old living at home. J Epidemiol Community Health.

[16] Robinson S, Syddall H, Jameson K, Batelaan S, Martin H, Dennison EM, Cooper C, Sayer AA, Group HS (2009). Current patterns of diet in community-dwelling older men and women: results from the Hertfordshire Cohort Study. Age Ageing.

[17] Office of Population Censuses and Surveys (1990) Standard occupational classification, Vol 1 Structure and definition of major, minor and unit groups. HMSO. London

[18] Genant HK, Wu CY, van KC, Nevitt MC (1993). Vertebral fracture assessment using a semiquantitative technique. J Bone Miner Res.

[19] Boutroy S, Bouxsein ML, Munoz F, Delmas PD (2005). In vivo assessment of trabecular bone microarchitecture by high-resolution peripheral quantitative computed tomography. J Clin Endocrinol Metab.

[20] Pauchard Y, Liphardt A-M, Macdonald HM, Hanley DA, Boyd SK (2012). Quality control for bone quality parameters affected by subject motion in high-resolution peripheral quantitative computed tomography. Bone.

[21] MacNeil JA, Boyd SK (2007). Accuracy of high-resolution peripheral quantitative computed tomography for measurement of bone quality. Med Eng Phys.

[22] Laib A, Hauselmann HJ, Ruegsegger P (1998). In vivo high resolution 3D-QCT of the human forearm. Technol Health Care.

[23] Khosla S, Riggs BL, Atkinson EJ, Oberg AL, McDaniel LJ, Holets M, Peterson JM, Melton LJ (2006). Effects of sex and age on bone microstructure at the ultradistal radius: a population-based noninvasive in vivo assessment. J Bone Miner Res.

[24] Biver E, Durosier-Izart C, Chevalley T, van Rietbergen B, Rizzoli R, Ferrari S (2018). Evaluation of radius microstructure and areal bone mineral density improves fracture prediction in postmenopausal women. J Bone Miner Res.

[25] Boutroy S, Van Rietbergen B, Sornay-Rendu E, Munoz F, Bouxsein ML, Delmas PD (2008). Finite element analysis based on in vivo HR-pQCT images of the distal radius is associated with wrist fracture in postmenopausal women. J Bone Miner Res.

[26] Burghardt AJ, Buie HR, Laib A, Majumdar S, Boyd SK (2010). Reproducibility of direct quantitative measures of cortical bone microarchitecture of the distal radius and tibia by HR-pQCT. Bone.

[27] Christen D, Melton LJ, Zwahlen A, Amin S, Khosla S, Muller R (2013). Improved fracture risk assessment based on nonlinear micro-finite element simulations from HRpQCT images at the distal radius. J Bone Miner Res.

[28] Seeman E (2002). Pathogenesis of bone fragility in women and men. Lancet.

[29] Langsetmo L, Peters KW, Burghardt AJ, Ensrud KE, Fink HA, Cawthon PM, Cauley JA, Schousboe JT, Barrett-Connor E, Orwoll ES (2018). Volumetric bone mineral density and failure load of distal limbs predict incident clinical fracture independent HR-pQCT BMD and failure load predicts incident clinical fracture of FRAX and clinical risk factors among older men. J Bone Miner Res.

[30] Nishiyama KK, Macdonald HM, Hanley DA, Boyd SK (2013). Women with previous fragility fractures can be classified based on bone microarchitecture and finite element analysis measured with HR-pQCT. Osteoporos Int.

[31] Sundh D, Mellstrom D, Nilsson M, Karlsson M, Ohlsson C, Lorentzon M (2015). Increased cortical porosity in older men with fracture. J Bone Miner Res.

[32] Sundh D, Nilsson AG, Nilsson M, Johansson L, Mellstrom D, Lorentzon M (2017). Increased cortical porosity in women with hip fracture. J Intern Med.

[33] Szulc P, Boutroy S, Chapurlat R (2018). Prediction of fractures in men using bone microarchitectural parameters assessed by high-resolution peripheral quantitative computed tomography-the prospective STRAMBO Study. J Bone Miner Res.

