# Supplementary material for: Cluster Analysis of Finite Element Analysis and Bone Microarchitectural Parameters Identifies Phenotypes with High Fracture Risk
Source: Calcif Tissue Int. 2019 Jun 11;105(3):252–62. doi: 10.1007/s00223-019-00564-7 (PMC6694037; doi:10.1007/s00223-019-00564-7)
Supplement: Supplementary file 1 — Supplementary material 1 (DOCX 15 kb) [file 223_2019_564_MOESM1_ESM.docx]

**Article title:** Cluster analysis of finite element analysis and bone microarchitectural parameters identifies phenotypes with high fracture risk

**Journal:** Calcified Tissue International and Musculoskeletal Research

**Authors**: LD Westbury, C Shere, MH Edwards, C Cooper (corresponding author), EM Dennison, KA Ward

**Affiliations and e-mail address for corresponding author**:

MRC Lifecourse Epidemiology Unit, University of Southampton, Southampton, UK

NIHR Southampton Biomedical Research Centre, University of Southampton and University Hospital Southampton NHS Foundation Trust, Southampton, UK

NIHR Oxford Biomedical Research Centre, University of Oxford, Oxford, UK

cc@mrc.soton.ac.uk

| **Appendix 1 Sex-specific Pearson correlations between bone parameters in receiver operating characteristic analysis** | | | |
| --- | --- | --- | --- |
|  |  |  |  |
| **Men** | Femoral neck aBMD | Total area | Cortical thickness |
| Total area | -0.02 |  |  |
| Cortical thickness | 0.27 | -0.51 |  |
| Trabecular separation | -0.46 | 0.05 | -0.27 |

| **Women** | Femoral neck aBMD | Cortical area | Cortical porosity | Trabecular density | Trabecular thickness | Failure load | Young modulus |
| --- | --- | --- | --- | --- | --- | --- | --- |
| Cortical area | 0.40 |  |  |  |  |  |  |
| Cortical  porosity | -0.06 | 0.16 |  |  |  |  |  |
| Trabecular  density | 0.51 | 0.34 | 0.34 |  |  |  |  |
| Trabecular  thickness | 0.35 | 0.22 | 0.24 | 0.78 |  |  |  |
| Failure load | 0.60 | 0.69 | 0.21 | 0.76 | 0.65 |  |  |
| Young  modulus | 0.50 | 0.62 | 0.15 | 0.70 | 0.71 | 0.81 |  |
| Von Mises stresses (trabecular) | 0.25 | 0.01 | 0.31 | 0.64 | 0.81 | 0.55 | 0.58 |
